# Brace treatment in juvenile idiopathic scoliosis: a prospective study with outcomes in agreement with SRS committee on bracing and nonoperative management standardization criteria

**DOI:** 10.1186/1748-7161-8-S2-O48

**Published:** 2013-09-18

**Authors:** Aulisa Angelo Gabriele, Vincenzo Guzzanti, Francesco Falciglia, Emanuele Marzetti, Marco Peruzzi, Lorenzo Aulisa

**Affiliations:** 1 Orthopaedic Department, Children's Hospital Bambino Gesù, Institute of Scientific Research, Rome, Italy

## Background

Based on age of onset, severity and evolutivity, the purpose and use of conservative methods to treat juvenile idiopathic scoliosis (JIS) is a source of great debate. The different clinical experiences highlight difficulties in applying a conservative treatment to patients with JIS, characterized by inevitable evolutivity, who are effectively facing a long growing period . 

## Purpose

The purpose of the present prospective study was to determine the effectiveness of conservative treatment in patients diagnosed with JIS. 

## Methods

A total of 1,238 individuals who were treated for JIS between 1995 and 2012 fulfilled the inclusion criteria (age between 4-10 years, full-time prescription), with 163 patients treated with progressive action short brace, Lyon brace and Milwaukee. Of these, 113 patients had a definite outcome, 27 abandoned treatment and 23 are still in treatment. The minimum duration of follow-up was 24 months. Antero-posterior radiographs were used to estimate the curve magnitude (CM) and the torsion of the apical vertebra (TA) at five points in time: beginning of treatment (t1), one year after the beginning of treatment (t2), intermediate time between t1 and t4 (t3), end of weaning (t4)and 2-year minimum follow-up from t4 (t5). Three outcomes were distinguished in agreement with Scoliosis Research Society criteria: curve correction, curve stabilization and curve progression. Moreover, results were evaluated according to compliance, dividing patients into five subgroups. Statistical analyses were performed with GraphPad Prism 6. 

## Results

The results of our study showed that, of the 113 patients with a definite outcome, the CM mean value was 30.55 ± 5.16 SD at t1 and 21.9 ± 7.65 SD at t5. TA was 13.58 ± 6.14 SD at t1 and 8.95 ± 5.82 at t5. The variations between measures of Cobb and Perdriolle degrees between CM t5-t1 and TA t5-t1 were statistically significantly different. Curve correction was accomplished in 88 patients (77.8%), whereas a curve stabilization was obtained in 18 patients (15.9%). Seven patients (6.19%) had a curve progression; of these, four (3.5%) were recommended for surgery. Of the 26 patients who abandoned treatment, at the time of abandonment (12.4 years of age) they had achieved curve correction in 19 cases (73.0%), curve stabilization in five cases (19.2%) and curve progression in three cases (11.5%). Of these patients, who were reviewed at the end of their growing periods, four have undergone surgery. In addition, there was a statistically significant correlation between compliance and result from t1-t5 with an interaction of 3.43 P <0.0001. 

## Conclusions and discussion

Our study confirmed that conservative treatment with bracing is highly effective in treating JIS, with most patients achieving a complete curve correction and only 4.9% of patients requiring surgery. In addition, the study confirmed that full-time bracing and patient compliance is essential to obtaining positive results. 
